# The dipeptide Phe-Phe amide attenuates signs of hyperalgesia, allodynia and nociception in diabetic mice using a mechanism involving the sigma receptor system

**DOI:** 10.1186/1744-8069-7-85

**Published:** 2011-10-31

**Authors:** Masahiro Ohsawa, Anna Carlsson, Megumi Asato, Takayuki Koizumi, Yuki Nakanishi, Rebecca Fransson, Anja Sandström, Mathias Hallberg, Fred Nyberg, Junzo Kamei

**Affiliations:** 1 Department of Pathophysiology & Therapeutics, School of Pharmacy and Pharmaceutical Sciences, Hoshi University, 4-41, Ebara 2-chome, Shinagawa-ku, Tokyo 142-8501, Japan; 2 Department of Pharmaceutical Biosciences, Uppsala University, P.O. Box 591, S-751 24 Uppsala, Sweden; 3 Department of Medicinal Chemistry, Uppsala University, P.O. Box 574, SE-751 23 Uppsala, Sweden

**Keywords:** Allodynia, Antinociception, Diabetes, Hyperalgesia, Opioid receptors, Phe-Phe amide, σ_1 _receptor, Substance P_1-7_

## Abstract

**Background:**

Previous studies have demonstrated that intrathecal administration of the substance P amino-terminal metabolite substance P_1-7 _(SP_1-7_) and its C-terminal amidated congener induced antihyperalgesic effects in diabetic mice. In this study, we studied a small synthetic dipeptide related to SP_1-7 _and endomorphin-2, i.e. Phe-Phe amide, using the tail-flick test and von Frey filament test in diabetic and non-diabetic mice.

**Results:**

Intrathecal treatment with the dipeptide increased the tail-flick latency in both diabetic and non-diabetic mice. This effect of Phe-Phe amide was significantly greater in diabetic mice than non-diabetic mice. The Phe-Phe amide-induced antinociceptive effect in both diabetic and non-diabetic mice was reversed by the σ_1 _receptor agonist (+)-pentazocine. Moreover, Phe-Phe amide attenuated mechanical allodynia in diabetic mice, which was reversible by (+)-pentazocine. The expression of spinal σ1 receptor mRNA and protein did not differ between diabetic mice and non-diabetic mice. On the other hand, the expression of phosphorylated extracellular signal-regulated protein kinase 1 (ERK1) and ERK2 proteins was enhanced in diabetic mice. (+)-Pentazocine caused phosphorylation of ERK1 and ERK2 proteins in non-diabetic mice, but not in diabetic mice.

**Conclusions:**

These results suggest that the spinal σ_1 _receptor system might contribute to diabetic mechanical allodynia and thermal hyperalgesia, which could be potently attenuated by Phe-Phe amide.

## Background

Diabetes is a global disease with an estimated worldwide prevalence of 2.8% in 2000, and this is predicted to climb to 4.4% in 2030 [1]. Diabetic neuropathy is seen in about 60% of all diabetic patients [2]. While the symptoms of diabetic polyneuropathy include hyperalgesia (hypersensitivity to noxious stimuli), hypoalgesia (loss of pain sensation) is also possible [3]. This pain is poorly relieved by opiates and the treatment regimen is usually based on the use of antiepileptics and antidepressants, which often have inadequate effects and are associated with a high prevalence of side effects [4]. There is a great need for new strategies for the treatment of diabetic neuropathy.

We recently demonstrated that substance P_1-7 _(SP_1-7_; H-Arg-Pro-Lys-Pro-Gln-Gln-Phe-OH), administered spinally, could attenuate thermal hyperalgesia in diabetic mice [5]. SP_1-7 _is formed from substance P (SP; H-Arg-Pro-Lys-Pro-Gln-Gln-Phe-Phe-Gly-Leu-Met-NH_2_). SP was discovered as a neuropeptide by Von Euler and Gaddum in 1931 [6] and is involved in pain signaling, peripheral inflammation and the maintenance of hyperalgesia [7]. SP is enzymatically degraded into several fragments, some of which retain their biological activity [8-10]. The main N-terminal fragment, SP_1-7_, exerts several effects that are opposite those of SP, e.g. the heptapeptide has antinociceptive [11], anti-inflammatory [12] and antihyperalgesic effects [5]. In addition, the heptapeptide attenuates several withdrawal signs in morphine-dependent rodents [13,14] and the development of morphine tolerance [13]. These effects are mediated through a specific receptor for SP_1-7_, which was detected in the rat and mouse spinal cord [15,16] and the rat ventral tegmental area [17], and is distinct from any of the known opioid and tachykinin receptors.

We previously reported that the σ_1 _receptor might be involved in the effect of SP_1-7 _and its amidated analogue, SP_1-7_-NH_2 _[5,18,19]. σ_1 _receptor ligands have poor affinity for the SP_1-7 _binding site [19] which may imply that SP_1-7 _has a downstream, rather than a direct, effect on the σ_1 _receptor. On the other hand, the effects of SP_1-7 _on σ_1 _receptors have been reported previously [20,21] and the σ_1 _receptor is interesting to study since it has been proposed to be involved in intracellular signaling cascades that lead to pain hypersensitivity [22].

We previously performed a thorough structure-activity relationship study of SP_1-7 _involving an alanine-scan and truncations as well as C- and N-terminal modification of the heptapeptide. Amidation of the heptapeptide increased its affinity for the SP_1-7 _binding sites [23] and produced a stronger effect than the native heptapeptide when used in behavioral tests [18,19]. The C-terminal part of SP_1-7_, especially the phenylalanine at position seven, is most essential for its binding. This finding, together with the knowledge that endomorphin-2 (EM-2; H-Tyr-Pro-Phe-Phe-NH_2_) binds to the SP_1-7 _binding site, resulted in the development of low-molecular, peptidergic ligands [24]. We discovered that the dipeptide H-Phe-Phe-NH_2 _(Phe-Phe amide) had the same affinity for the SP_1-7 _binding site as the endogenous heptapeptide. Therefore, the present study was designed to examine the ability of this dipeptide to attenuate allodynia and thermal hyperalgesia in diabetic mice.

## Results

### Effect of Phe-Phe amide on the thermal nociceptive threshold in diabetic and non-diabetic mice

**Figure 1:**
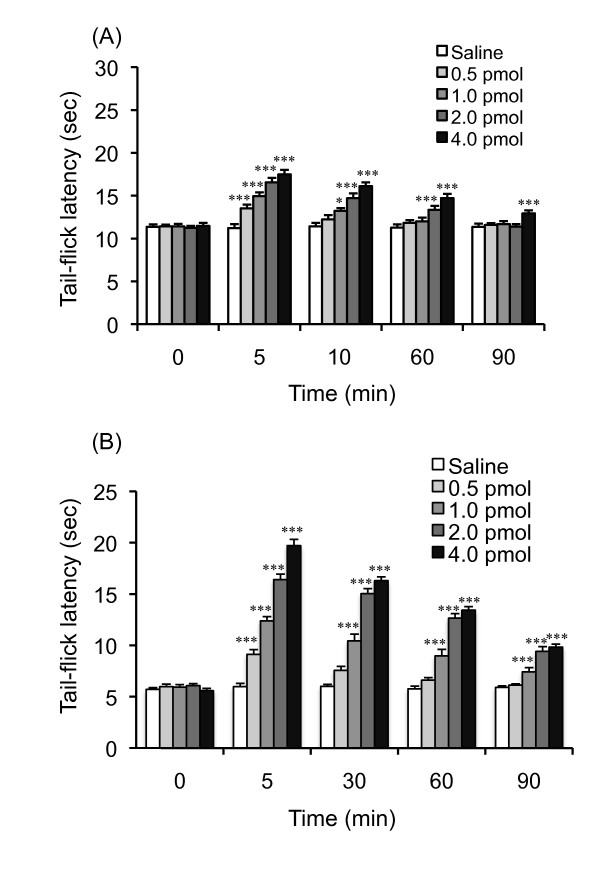
**Time-response curve for the intrathecal injection of Phe-Phe amide on the tail-flick latency in non-diabetic (A) and diabetic (B) mice**. The nociceptive threshold was determined by the tail-flick test 5, 30 60 and 90 min after injection of the dipeptide. Each point represents the mean with S.E.M. for 10-12 mice. *P < 0.05 and ***P < 0.001 vs. saline-treated group.

The baseline tail-flick latencies in diabetic mice were shorter than those in non-diabetic mice, indicating that diabetic mice have a reduced pain threshold (Figures 1A and 1B). As shown in Figure 1A, i.t. administration of Phe-Phe amide dose-dependently increased the tail-flick latencies in non-diabetic mice. Two-way ANOVA indicated a significant main effect of Phe-Phe amide treatment (F_4, 250 _= 46.59, P < 0.001), time (F_4,250 _= 58.8, P < 0.001), and the interaction between Phe-Phe amide treatment and time (F_16,250 _= 6.93, P < 0.001). A dose-dependent increase in tail-flick latency was seen in diabetic mice, but with greater potency (Figure 1B). Two-way ANOVA indicated a significant main effect of Phe-Phe amide treatment (F_4,230 _= 382.55, P < 0.001), time (F_4,230 _= 338.56, P < 0.001), and the interaction between Phe-Phe amide treatment and time (F_16, 230 _= 45.71, P < 0.001). Since the antinociceptive effect of Phe-Phe amide was more potent in diabetic mice, the ability of Phe-Phe amide to produce an increase in the tail-flick latency in diabetic mice is greater than that in non-diabetic mice.

### Effects of opioid receptor antagonists on Phe-Phe amide-induced antinociception in diabetic and non-diabetic mice

**Figure 2:**
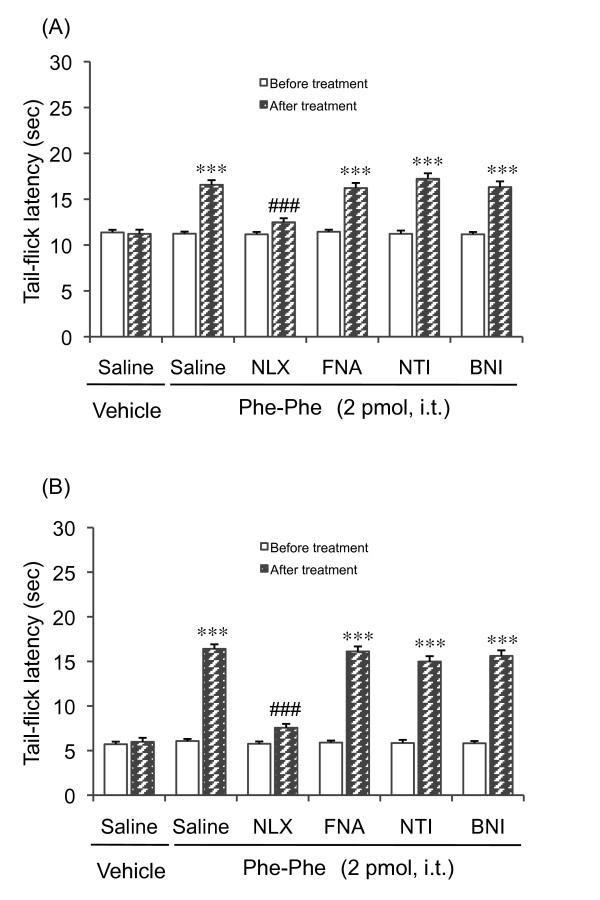
**Effects of opioid receptor antagonists on Phe-Phe amide (2 pmol, i.t.)-induced antinociception in non-diabetic (A) and diabetic (B) mice**. Naloxone (NLX, 1 mg/kg i.p) and naltrindole (NTI, 3 mg/kg, s.c.) were administered 30 min before the injection of Phe-Phe amide. β-Funaltrexamine (β-FNA, 20 mg/kg, s.c.) and nor-binaltorphimine (BNI, 20 mg/kg, s.c.) were administered 24 h before injection of the Phe-Phe amide. Each column represents the mean with S.E.M. for 6-11 mice. ***P < 0.001 vs. respective before Phe-Phe amide-treatment group; ###P < 0.001 vs. saline-pretreated Phe-Phe amide group (Bonferroni test).

To investigate whether the opioid system is involved in the effect seen with Phe-Phe amide, we examined the effects of opioid receptor antagonists on the prolongation of the tail-flick latency seen after the administration of 2 pmol Phe-Phe amide. This dose was chosen according to the results of the dose-response study, where it was shown to produce an evident antinociceptive response in both diabetic and non-diabetic mice. Pretreatment with the non-selective opioid receptor antagonist naloxone (1 mg/kg, i.p.) inhibited the Phe-Phe amide-induced increase in the tail-flick latency in non-diabetic mice (Figure 2A) as well as in diabetic mice (Figure 2B). In contrast to naloxone, neither μ-, δ-, nor κ-opioid receptor antagonists had any effect on the Phe-Phe amide-induced prolongation of the tail-flick latency (Figure 2A and 2B).

### Involvement of σ-receptors in Phe-Phe amide-induced antinociception in diabetic and non-diabetic mice

**Figure 3:**
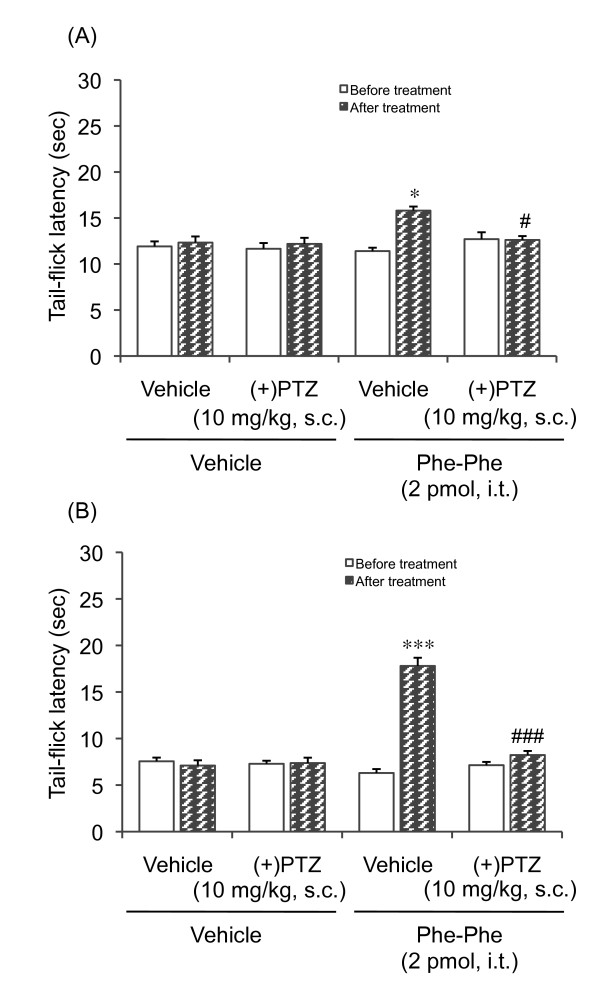
**Effect of the σ_1_-receptor agonist (+)-pentazocine [(+)PTZ, 10 mg/kg, s.c] on the Phe-Phe amide (2 pmol, i.t.)-induced prolongation of the tail-flick latency in non-diabetic and diabetic mice**. (+)PTZ was administered subcutaneously 30 min prior to the administration of Phe-Phe amide. Each column represents the mean with S.E.M. for 10 mice. *P < 0.05, ***P < 0.001 vs. respective before-treatment group; #P < 0.05, ###*P *< 0.001 vs. vehicle-pretreated group (Bonferroni test).

We recently demonstrated that the antihyperalgesic effects induced by both SP_1-7 _and its analogue SP_1-7_-NH_2 _in diabetic mice may involve σ_1 _receptors. Therefore, we also evaluated the possible involvement of the σ_1 _receptor by examining the effect of the agonist (+)-pentazocine on Phe-Phe amide-induced antinociception in diabetic and non-diabetic mice. Pretreatment with (+)-pentazocine attenuated the Phe-Phe amide-induced prolongation of the tail-flick latency in both non-diabetic (Figure 3A) and diabetic mice (Figure 3B).

### Effect of Phe-Phe amide on mechanical allodynia in diabetic mice

**Figure 4:**
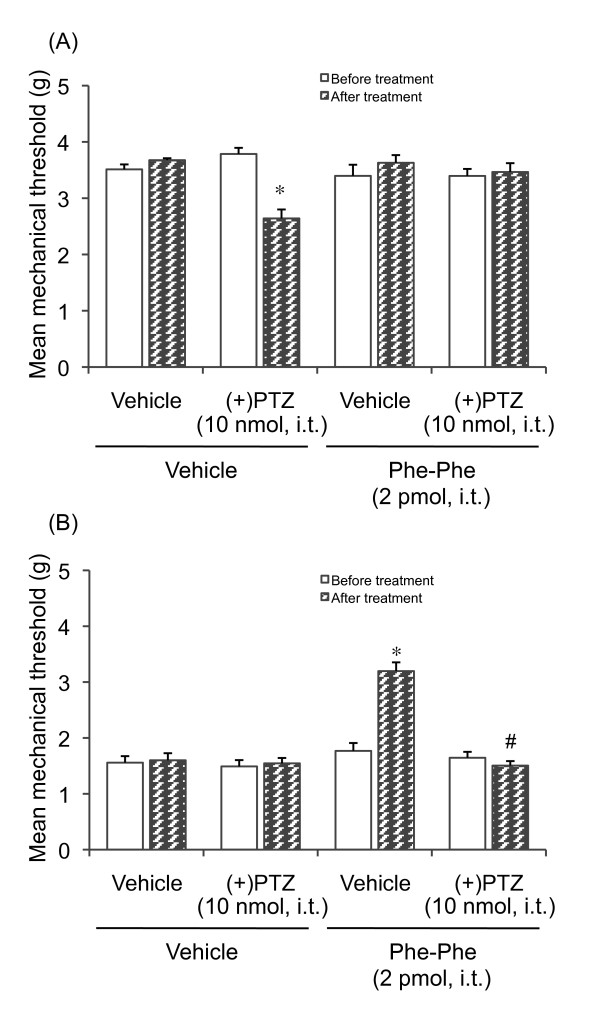
**Effect of Phe-Phe amide (2 pmol, i.t.) on the mechanical threshold in diabetic and non-diabetic mice**. The nociceptive threshold was determined by a von Frey filament test 30 min after dipeptide injection. (+)-Pentazocine [(+)PTZ, 10 nmol, i.t] was administered 10 min prior to the injection of Phe-Phe amide. Each point represents the mean with S.E.M. for 7-11 mice. *P < 0.05 vs. respective before-treatment group; #*P *< 0.001 vs. vehicle-pretreated group (Bonferroni test).

Phe-Phe amide produced a significant increase in the thermal threshold in diabetic and non-diabetic mice. In the next step of the experiment, we investigated the effect of Phe-Phe amide on the mechanical threshold in diabetic and non-diabetic mice. As shown in Figure 4, the mechanical threshold in diabetic mice was lower than that in non-diabetic mice, indicating that diabetic mice exhibit mechanical allodynia (Figure 4A and 4B). I.t. administration of Phe-Phe amide (2 pmol) did not affect the mechanical threshold in non-diabetic mice (Figure 4A). In contrast to non-diabetic mice, the decrease in the mechanical threshold in diabetic mice was reversed by treatment with Phe-Phe amide, an effect which in turn was attenuated by i.t. pretreatment with (+)-pentazocine (Figure 4B). Although i.t. treatment with Phe-Phe amide did not affect the mechanical threshold in non-diabetic mice, i.t. administration of (+)-pentazocine decreased the mechanical threshold in non-diabetic mice, which was reversed by i.t. treatment with Phe-Phe amide (Figure 4A).

**Figure 5:**
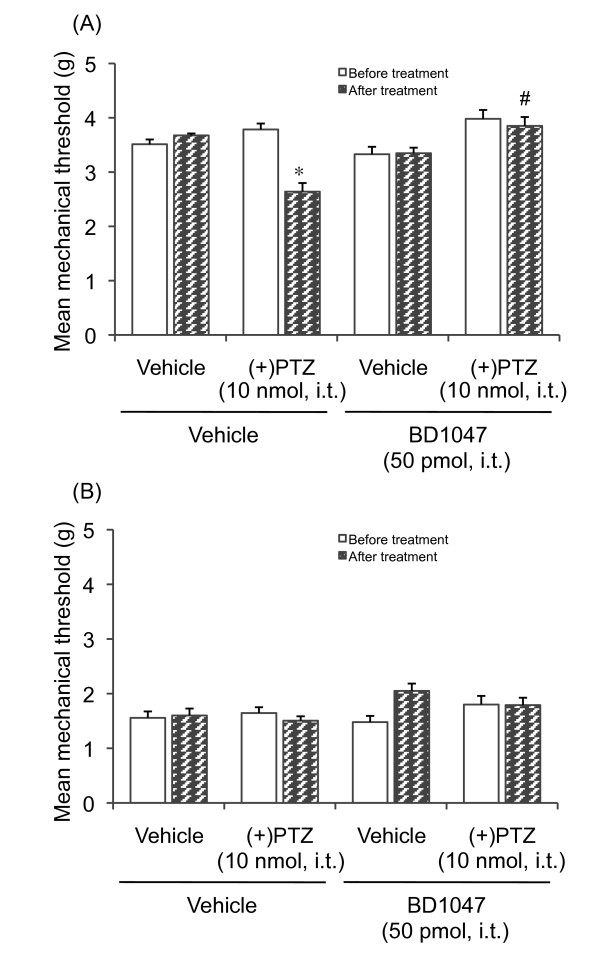
**Effect of the σ_1_-receptor antagonist BD1047 on (+)-pentazocine [(+)PTZ, 10 nmol, i.t.]-induced lowering of the mechanical threshold in non-diabetic and diabetic mice**. BD1047 was injected i.t. 10 min prior to the administration of (+)PTZ. Each column represents the mean with S.E.M. for 10 mice. *P < 0.05 vs. respective before-treatment group; #*P *< 0.05 vs. vehicle-pretreated group (Bonferroni test).

This i.t.-administered (+)-pentazocine-induced decrease in the mechanical threshold in non-diabetic mice suggests that the σ_1 _receptor may play a role in mechanical allodynia. To confirm this possibility, the effect of the σ_1 _receptor antagonist BD1047 on the (+)-pentazocine-induced decrease in the mechanical threshold in non-diabetic mice was examined. I.t. pretreatment with BD1047 completely reversed the decrease in the mechanical threshold in (+)-pentazocine-treated non-diabetic mice (Figure 5A). I.t. treatment with BD1047 slightly, but not significantly, increased the mechanical threshold in diabetic mice, while the mechanical threshold in non-diabetic mice was not affected (Figure 5B).

### Expression of σ_1 _receptor mRNA and proteins in the spinal cords of diabetic and non-diabetic mice

**Figure 6:**
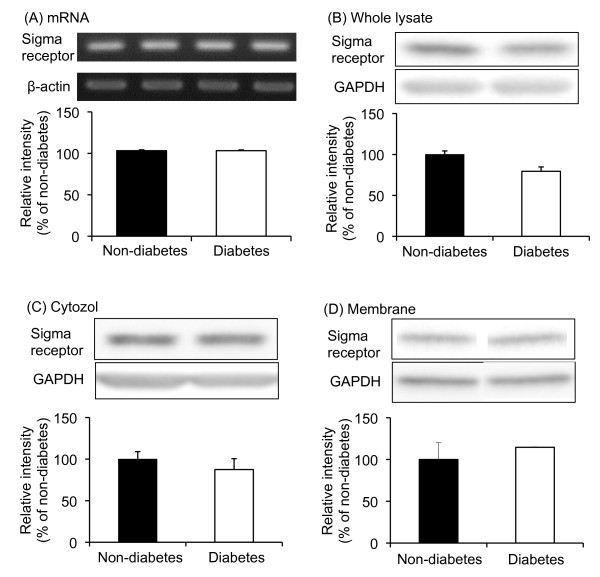
**σ_1 _Receptor mRNA (A), and protein (B, C, D) expression in the spinal cords of diabetic and non-diabetic mice**. Protein expression was evaluated in the whole lysate (B), cytosol fraction (C), and membrane fraction (D). Immunoblots are from an experiment that is of 4 similar experiments. Each column represents the mean ± S.E.M. of 4 separate experiments.

The behavioral studies strongly suggested that the spinal σ_1 _receptor system is enhanced in diabetic mice. Moreover, the potent antinociceptive and antiallodynic effect of Phe-Phe amide in diabetic mice might be correlated with enhanced activity in the spinal σ_1 _receptor system. To clarify this possibility, the expression of σ_1 _receptor mRNA and protein was examined in the spinal cords of diabetic and non-diabetic mice. Reverse-transcription semi-quantitative PCR indicated that the level of σ_1 _receptor mRNA in the spinal cord in diabetic mice was not different from that in non-diabetic mice (Figure 6A). Moreover, the expression of the σ_1 _receptor protein in the spinal cord was unchanged in diabetic mice (Figure 6B). σ_1 _receptors have been shown to translocate from the mitochondrion-associated endoplasmic reticulum membrane to the plasma membrane, which leads to functional activation [25]. Therefore, the expression of the σ_1 _receptor in the cytosol and membrane fractions in the spinal cords of diabetic and non-diabetic mice was examined. The expression of σ_1 _receptors in the cytosol and membrane fraction of the spinal cord in diabetic mice was not different from that in non-diabetic mice (Figure 6C and 6D).

### Effect of (+)-pentazocine on the phosphorylation of ERK1 and ERK2 proteins in the spinal cords of diabetic and non-diabetic mice

**Figure 7:**
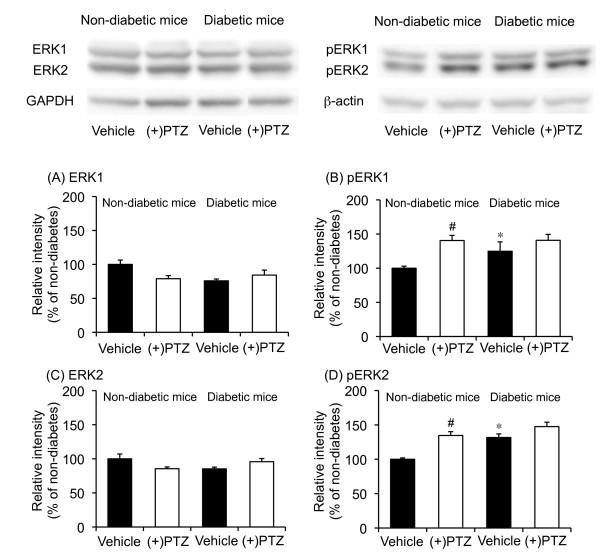
**Effects of (+)-pentazocine [(+)PTZ] on the expression of ERK1 (A), ERK2 (C), phosphorylated ERK1 (B), and phosphorylated ERK2 (D) protein in the spinal cords of diabetic and non-diabetic mice**. Spinal samples were collected 30 min after the i.t. administration of (+)PTZ. Immunoblots are from an experiment that is representative of 4 similar experiments. Each column represents the mean ± S.E.M of 4 separate experiments. *P < 0.05 vs. non-diabetic mice; #P < 0.05 vs. respective vehicle-treated group.

Since σ_1 _receptor expression was not changed in diabetic mice, the function of σ_1 _receptors in diabetic mice might be enhanced in the spinal cord. Therefore, we examined the effect of (+)-pentazocine on the phosphorylation of the extracellular signal-regulated protein kinase 1 (ERK1) and ERK2 proteins in the spinal cords of diabetic and non-diabetic mice. The expression of ERK1 and ERK2 proteins in the spinal cord of diabetic mice was not different than that in non-diabetic mice (Figure 7A and 7B). In contrast to the expression of ERK1 and ERK2 protein, the expression of phosphorylated ERK1 and ERK2 proteins was increased in the spinal cord of diabetic mouse compared to non-diabetic mice (Figure 7B and 7D). Treatment with (+)-pentazocine did not affect the expression of ERK1 and ERK2 protein in the spinal cords of diabetic and non-diabetic mice (Figure 7A and 7B). Treatment with (+)-pentazocine increased the phosphorylation of ERK1 and ERK2 in the spinal cord of non-diabetic mice (Figure 7A and 7B). In contrast to non-diabetic mice, phosphorylation of ERK1 and ERK2 was not observed in diabetic mouse spinal cord after treatment with (+)-pentazocine (Figure 7B and 7D).

## Discussion

The present results showed that the i.t. administration of Phe-Phe amide increased the tail-flick latency in diabetic and non-diabetic mice the same as larger SP-related peptides [5,18]. The Phe-Phe amide-induced increase in the tail-flick latency in diabetic mice was greater than that in non-diabetic mice. The antinociception elicited by the dipeptide in both diabetic and non-diabetic mice was completely blocked by the non-selective opioid receptor antagonist naloxone. However, none of the more selective μ-, δ- or κ- opioid receptor antagonists (β-funaltrexamine, naltrindole and nor-binaltorphimine, respectively) affected Phe-Phe amide-induced antinociception, suggesting that the antinociceptive effect of the dipeptide amide does not involve these receptors. Tsao and Su [26] previously reported that the (+)- stereoisomer of naloxone may interact with the σ_1 _receptor. We recently found that SP_1-7 _produced a σ_1 _receptor-sensitive antinociception in diabetic and non-diabetic mice [5], and that Phe-Phe amide exhibited high affinity for the specific binding site of SP_1-7 _[24]. We observed in the present study that i.t. pretreatment with (+)-pentazocine attenuated the Phe-Phe amide-induced antinociception in diabetic and non-diabetic mice, suggesting that the σ_1 _receptor may be involved in the antinociceptive effect of Phe-Phe amide.

The σ_1 _receptor system has previously been shown to influence the mechanical pain threshold [22]. We observed that the mechanical threshold in diabetic mice was lower than that in non-diabetic mice. Treatment with Phe-Phe amide increased the mechanical threshold in diabetic mice, but not non-diabetic mice. This Phe-Phe amide-induced antiallodynic effect in diabetic mice was attenuated by pretreatment with (+)-pentazocine, which again suggests that the spinal σ_1 _receptor system may be involved. Interestingly, (+)-pentazocine by itself decreased the mechanical threshold in non-diabetic mice. This (+)-pentazocine-induced mechanical allodynia was attenuated by pretreatment with the σ_1 _receptor antagonist BD1047 and Phe-Phe amide, suggesting that the activation of spinal σ_1 _receptors causes mechanical allodynia. It has been reported that spinal σ_1 _receptor activation enhances the N-methyl-D-aspartate (NMDA)-induced nociceptive response [27]. Moreover, mice that lack the σ_1 _receptor did not exhibit any mechanical allodynia after nerve ligation [22], which confirms that activation of the spinal σ_1 _receptor system might be involved in mechanical allodynia. On the other hand, i.t. administration of (+)-pentazocine in diabetic mice did not affect the mechanical threshold. A possible explanation for this finding is that the spinal σ_1 _receptor-mediated system is already activated in diabetic mice. This possibility is supported by the finding that i.t. pretreatment with the σ_1 _receptor antagonist BD1047 slightly, but not significantly, increased the mechanical threshold in diabetic mice.

I.t. administration of (+)-pentazocine produced mechanical allodynia in non-diabetic mice (see Figure 4A) but did not affect the thermal nociceptive threshold (Figure 3A). This suggests the differential modulation of nociceptive pathways by σ_1 _receptors depending on the stimulus quality and modality. This adds to previous findings that different receptor systems and mechanisms are involved in diverse sensory abnormalities, such as mechanical versus thermal pain [22,28-31].

We also examined the expression of the σ_1 _receptor gene transcript and protein in the spinal cord of both diabetic and non-diabetic mice, and did not find any differences. One of the few studies on the σ_1 _receptor in diabetic mice demonstrated that there were no differences in the expression of the σ_1 _receptor gene transcript and protein in retinal ganglion cells between diabetic and non-diabetic mice [32]. Furthermore, a receptor binding study has indicated a decrease in σ_1 _receptor density in the brains of long-term diabetic rats, while relatively short-term diabetic rats did not show any significant differences [33].

Since the expression of the spinal σ_1 _receptor mRNA and protein is not changed in diabetic mice, the function of σ_1 _receptors might be affected. Activation of the σ_1 _receptor increases the intracellular Ca^2+ ^concentration by potentiating Ca^2+ ^entry at the plasma membrane and Ca^2+ ^mobilization from endoplasmic stores. Previously, it has been reported that σ_1 _receptors are functionally coupled to the NMDA receptor [34-38] and regulate intracellular Ca^2+ ^concentration through phospholipase C and IP_3 _[39,40]. Ca^2+ ^entry into neurons through the NMDA receptor or mobilization through IP_3 _may initiate the intracellular ERK signaling cascade in spinal dorsal horn neurons [41], thus contributing to central sensitization. The present study indicates that the expression levels of phosphorylated ERK1 and ERK2 are increased in diabetic mice compared to non-diabetic mice, while the expression levels of ERK1 and ERK2 protein are not changed. These results suggest that spinal ERK signaling is already activated in the spinal cord of diabetic mice. Moreover, i.t. treatment with (+)-pentazocine increased the phosphorylation of ERK1 and ERK2 proteins in the spinal cord of non-diabetic mice, but not diabetic mice. This observation suggests that the stimulation of spinal σ_1 _receptors produces the activation of ERK signaling. A previous report indicated that ERK on the ipsilateral side of the spinal cord dorsal horn was activated in nerve-ligated mice [42]. This ERK activation in the spinal cord after nerve ligation was not observed in σ_1 _receptor knockout mice [22], which supports our present finding that σ_1 _receptors are responsible for the regulation of ERK signaling in diabetic mice. Since (+)-pentazocine did not increase the phosphorylation of spinal ERK protein in diabetic mice, the increased phosphorylation of ERK1 and ERK2 protein in the spinal cord may be, at least in part, due to the enhancement of σ_1 _receptor-mediated functions.

The present results do not clarify the action site of Phe-Phe amide. Our recent findings clearly indicated that Phe-Phe amide has very high affinity for the SP_1-7 _binding site [24], which is distinct from the neurokinin receptors, NK1 and NK3 [15,24]. Our previous studies suggested that SP_1-7 _might be related to the σ receptor system, since a σ1 receptor agonist could reverse the effect of SP_1-7 _on hyperalgesia and naloxone-precipitated morphine withdrawal signs [5,19]. However, (+)-pentazocine has very low affinity for the SP_1-7 _binding site [19], suggesting that SP_1-7 _and its analogues modulate the effect of the σ_1 _receptor system rather than directly acting as ligands for σ_1 _receptors.

## Conclusions

The present study suggests that the antinociceptive and antiallodynic effects induced by Phe-Phe amide occur through modulation of the spinal σ_1 _receptor system via the SP_1-7 _binding site. Furthermore, the spinal σ_1 _receptor system appears to contribute to the thermal hyperalgesia and mechanical allodynia seen in diabetic mice. Based on the present results, compounds that bind to a SP1-7-specific binding site, like Phe-Phe amide, might be attractive for the treatment of pain symptoms associated with diabetic neuropathy. Notably, this small synthetic dipeptide, with high affinity for the SP_1-7 _binding site, had a more pronounced *in vivo *effect than larger SP-related peptides. Since Phe-Phe amide has analgesic properties similar to those of SP_1-7 _as well as a smaller size, it is an interesting starting point for the development of new drugs to relieve neuropathic pain.

## Methods

This study was carried out in accordance with the Declaration of Helsinki and/or with the guide for the committee on the care and use of laboratory animals of Hoshi University, Tokyo, which is accredited by the Ministry of Education, Science, Sports and Culture.

### Animals

Male 4-week-old ICR mice (Tokyo Animal Laboratory Inc, Tokyo) weighing about 20 g at the start of the experiment were used. The mice had free access to food and water and were housed five per cage in an animal room that was maintained at 24 ± 1°C with a 12-h dark/light cycle. All behavioral experiments were performed between 10:00 and 19:00 each day.

### Induction of diabetes with streptozotocin

Mice were rendered diabetic by an intravenous injection of streptozotocin (200 mg/kg) prepared in a 0.1 N citrate buffer at pH 4.5. Age-matched animals were injected with vehicle alone. Experiments were conducted two weeks after the administration of streptozotocin. Animals with a serum glucose level exceeding 400 mg/dl were considered diabetic.

### Assessment of thermal hyperalgesia

The antihyperalgesic response was evaluated using the tail-flick test (KN-205E Thermal Analgesimeter, Natume, Tokyo, Japan) as described by D'amour and Smith [43]. Briefly, the mouse was gently restrained in a tube. The tail was positioned in a groove underneath a 50 W projection bulb with the dorsal part of the tail facing the light bulb. The light and timer were monitored with the same switch. Twitching or movement of the tail is a typical response elicited by heat. When this occurred, light reached a photocell and the light and timer were switched off. Latencies were determined as the mean of two trials. The voltage of a 50 W projection lamp was set to 50 V [44], which gave a baseline value in non-diabetic animals of 10-14 s. The cut-off time was set to 30 s to prevent injury to the tail. Tail-flick latencies were measured 5, 30, 60 and 90 min after i.t. injection of Phe-Phe amide.

### Assessment of tactile allodynia

Mechanical sensitivity was determined by probing the plantar surface of the hind paw (von-Frey test) with a calibrated plastic filament of a dynamic plantar aesthesiometer purchased from Ugo Basile (Comerio, Italy). Force was applied to the hind paw at a rate of 0.25 g/s; the final force when paw withdrawal was observed was measured automatically (mechanical threshold). A maximal cut-off of 5 g was used to prevent tissue damage. A significant decrease in the mechanical threshold after the induction of diabetes compared with that in vehicle-treated animals was considered mechanical allodynia. The mechanical threshold was determined as the average of two measurements per mouse. Values were obtained before and 30 minutes after drug administration.

### Intrathecal injections

Phe-Phe amide and σ ligands were administered by i.t. injection as described by Hylden and Wilcox [45] using a 25 μl Gastight^® ^syringe (Hamilton, USA) and a BD Precisionglide^® ^30G 1/2 inch needle (Becton Dickinson, USA). The mouse was restrained manually and the needle was inserted between the L5 and L6 vertebrae. This site is near the end of the spinal cord and minimizes the risk of spinal damage [45].

### Western blot

The spinal cord was quickly removed following decapitation to evaluate σ_1 _receptor proteins. To measure the phosphorylation of ERK protein, decapitation was performed 30 min after treatment with (+)-pentazocine. The spinal cord was homogenized in ice-cold buffered sucrose solution containing 20 mM Tris-HCl (pH 7.4), 2 mM EDTA, 0.5 mM EGTA, and 1 mM phenylmethylsulfonyl fluoride plus 250 μg/ml leupeptin, 250 μg/ml aprotinin, and 0.32 M sucrose. The homogenate was then centrifuged at 1,000 × g for 10 min at 4°C, and the resulting supernatant was centrifuged at 9000 × g for 20 min at 4°C. To separate the cytosol and membrane fractions, the supernatant was ultracentrifuged at 100,000 × g for 60 min at 4°C. The resulting supernatant was retained as the cytosolic fraction. The pellet was resuspended in ice-cold Tris buffer (ice-cold buffer without sucrose) and ultracentrifuged at 100,000 × g for 60 min. The supernatant was discarded and the pellet was resuspended in ice-cold Tris buffer. The protein concentration was measured using a Bradford assay kit (Thermo Fisher Scientific Inc., Suwannee, GA). Cytosol and plasma membrane samples with the same amounts of protein were diluted with an equal volume of 2x electrophoresis sample buffer containing 2% SDS and 10% glycerol with 0.2 M dithiothreitol. Proteins (20 μg) were separated by size on 5-20% SDS-polyacrylamide gradient gel using the buffer system and transferred to nitrocellulose membranes in Tris-glycine buffer containing 25 mM Tris and 192 mM glycine. For immunoblot detection, the membranes were blocked in Tris-buffered saline (TBS) containing 5% non-fat dried milk or 1% non-fat dried milk with 0.1% Tween 20 (Bio-Rad Laboratories, Hercules, CA, USA) for 1 hr at room temperature with agitation. The membrane was immunoblotted overnight at 4°C with antibodies against σ_1 _receptor (1:500; Santa Cruz Biotechnology Inc., Santa Cruz, CA, USA), ERK1 (1:1000; Cell Signaling Technology Inc., Danvers, MA, USA), ERK2 (1:1000; Cell Signaling Technology Inc.), phosphorylated ERK1 (Cell Signaling Technology Inc.), and phosphorylated ERK2 (Cell Signaling Technology Inc.). The membrane was washed in TBS containing 0.05% Tween 20 (TTBS), and then incubated for 2 h at room temperature with horseradish peroxidase-conjugated goat anti-rabbit IgG or rabbit anti-goat IgG (Southern Biotechnology Associates, Inc., Birmingham, AL, USA) diluted 1:10,000 in TBS containing 5% nonfat dried milk or 1% non-fat dried milk with 0.1% Tween 20. The antigen-antibody peroxidase complex was finally detected by enhanced chemiluminescence (Pierce, Rockford, IL, USA) and visualized using a Light-Capture II imaging system (Atto Co., Tokyo, Japan). The intensity of the band was analyzed and semi-quantified by computer-assisted densitometry using the NIH imaging system. Each value for σ_1 _receptors in diabetic and non-diabetic mice was normalized by the respective value for the internal control GAPDH.

### Semi-quantification of reverse-transcription polymerase chain reaction (RT-PCR)

Total RNA was extracted from the mouse spinal cord using a FastPure RNA isolation kit (Takara Bio Inc., Shiga, Japan) according to the manufacturer's instructions. Total RNA was quantified by a spectrophotometer at A260. Next, cDNA was synthesized by a PrimeScript RT reagent kit (Takara Bio) with oligo dT primers. The mouse sigma1 receptor cDNA was amplified in 25 μl of PCR solution containing 0.8 mM MgCl_2_, 250 nM NTP mixture, and 0.5 units of platinum Taq DNA polymerase with synthesized primers (0.5 μM) corresponding to the mouse σ_1 _receptor cDNA (GenBankTM accession number XM136229). The primers used were as follows: 5'-primer, 5'-CAT TCG GGG CGA TAC TGG GC-3' (1-22); 3'-primer 5'-CCT GGG TAG AAG ACC TCA CTT TT -3'(311-332). Samples were heated to 94°C for 2 min, 94°C for 30 sec, 55°C for 1 min, and 72°C for 2 min for 35 cycles. The final incubation was at 72°C for 10 min. The mixture was run on 1.5% agarose gel electrophoresis with the indicated markers and primers for the internal standard β-actin. The agarose gel was stained with ethidium bromide and photographed with UV transillumination. The intensity of the band was analyzed and semi-quantified by computer-assisted densitometry using Image J. Data are expressed as a ratio to β-actin.

### Chemicals

The non-selective opioid receptor antagonist naloxone, the σ_1 _receptor agonist (+)-pentazocine, and the σ_1 _receptor antagonist BD1047 were purchased from Sigma Chemical Co (St. Louis, MO, USA). Phe-Phe amide was prepared by using solid-phase peptide synthesis. Standard Fmoc conditions were used and the protecting group was removed by 20% piperidine in DMF. The coupling procedure was performed in N, N-dimethylformamide (DMF), using N-[(1H-benzotriazole-1-yl)-(dimethylamino)methylene]-N-methylmethanaminium hexafluorophosphate N-oxide (HBTU) as a coupling reagent and N, N-diisopropylethylamine (DIEA) as a base. The dipeptide was cleaved from the resin by the addition of triethylsilane and 95% aqueous trifluoroactic acid (TFA), and purified by RP-HPLC to give Phe-Phe amide with purity above 99%. The selective μ-opioid receptor antagonist β-funaltrexamine, the selective δ-opioid receptor antagonist naltrindole, and the selective κ-opioid receptor antagonist nor-binaltorphimine were gifts from Toray Industries, Inc. (Kanagawa, Japan). (+)-Pentazocine was dissolved in a vehicle solution of 90% sterile saline (0.9% NaCl), 5% dimethylsulfoxide (DMSO), and 5% cremophore EL (Sigma), whereas all other drugs were dissolved in saline. Naloxone and naltrindole were injected intraperitoneally (i.p.) and subcutaneously (s.c), respectively, 30 min before injection of the peptide. (+)-Pentazocine was injected i.t. 10 min before the injection of Phe-Phe amide. β-Funaltrexamine and nor-binaltorphimine were injected s.c. 24 h before injection of the peptide. I.t. pretreatment with BD1047 was performed 10 min before the administration of (+)-pentazocine. The doses and routes for the administration of each antagonist were according to previous reports [5,46,47].

### Statistical analysis

Data are presented as the mean ± S.E.M. Differences between treatment groups were evaluated using one-way or two-way analysis of variance (ANOVA) followed by the Bonferroni-Dunn test. At all times, a level of probability of 0.05 or less (P < 0.05) was considered significant.

## List of abbreviations

Phe-Phe amide: Phenylalanine-phenylalanine amide; SP: substance P; EM-2: endomorphin-2; ANOVA: analysis of variance; i.p: intraperitoneal; i.t.: intrathecal; BD1047: N'-[2-(3,4-dichlorophenyl)ethyl]-N, N, N'-trimethylethane-1,2-diamine; ERK: extracellular signal-regulated protein kinase; NMDA: N-methyl D-aspartate; IP3: inositol 1,4,5-trisphosphate; NK: neurokinin

## Competing interests

The authors declare that they have no competing interests.

## Authors' contributions

MO carried out the behavioral and molecular studies, performed the statistical analysis, and participated in drafting the manuscript. AC, TK and YN carried out the behavioral studies. MA carried out the western blot study. RF and AS prepared Phe-Phe amide. MH edited the manuscript. FN supervised the project, coordinated the studies, and edited the manuscript. JK conceived of the project, designed and coordinated the studies, and drafted and edited the manuscript. All authors contributed to data interpretation, and have read and approved the final manuscript.
